# Interpreting musical solitude through practice-led research: a reflexive study of four vocal works

**DOI:** 10.3389/fpsyg.2026.1820211

**Published:** 2026-10-06

**Authors:** Yu Weng

**Affiliations:** Institute of Music, University College Sedaya International, Kuala Lumpur, Malaysia

**Keywords:** musical solitude, performance studies, practice-led research, score analysis, vocal repertoire

## Abstract

Understanding musical solitude in vocal performance requires tracing how musical and poetic observations inform embodied interpretive decisions across contrasting repertoires. This study examines four contrasting vocal works, Henry Purcell’s *O Solitude*, Claude Debussy’s *Le son du cor s’afflige vers les bois*, Alban Berg’s *Wir arme Leut!* from *Wozzeck*, and Ildebrando Pizzetti’s *Oscuro è il ciel*, to investigate how musical solitude is recognized, interpreted, and realized through reflexive artistic inquiry. A qualitative practice-led research design integrated score and text analysis, historical and musicological contextualization, embodied vocal experimentation, iterative recording comparison, and critical reflection. Poetic and dramatic texts were treated as integral components of the score, informing decisions about declamation, prosody, phrasing, breathing, diction, resonance, timbre, dynamics, and expressive timing. Comparative analysis suggested that musical solitude was realized through relationships among score, text, context, and vocal practice rather than through any isolated expressive feature. A final cross-case synthesis brought these understandings together under four repertoire-specific interpretive mechanisms: Exposure, Suspension, Fragmentation, and Restraint. Each mechanism names an interpretive relationship through which textual meaning informed vocal sound and artistic performance. These case-grounded formulations offer a vocabulary for examining interpretive decision-making, not a fixed classification of solitude. By showing how musical structure, poetic meaning, and embodied vocal practice mutually inform artistic inquiry, the study contributes to methodological understanding of practice-led research and offers a framework that may be tested or adapted in further studies of vocal interpretation.

## Introduction

Every musical performance requires interpretive decision-making (Rink, 2002). Although musical scores specify pitches, rhythms, dynamics, articulations, and other compositional instructions, they rarely determine how expressive qualities should be realized in performance. Interpretation therefore extends beyond the accurate reproduction of notation, requiring performers to negotiate relationships among compositional structure, historical knowledge, stylistic conventions, embodied experience, and artistic intention (Cook, 2013; Godlovitch, 1998; Small, 1998). Through this process, the expressive potential of a musical work is transformed into artistic performance.

Musicological research has long demonstrated that musical meaning emerges through the interaction of compositional organization, historical context, and performance rather than residing solely within the score (Cook, 1990; Kramer, 2002; Zbikowski, 2002; Clarke, 2005). Within this broader field, solitude has attracted increasing scholarly attention as both a cultural concept and an artistic phenomenon. Studies have shown how rhetorical emphasis, harmonic ambiguity, motivic organization, text setting, and formal design contribute to different representations of solitude across musical traditions (Meyer, 1956; Hatten, 2004; Monelle, 2006; Stern, 2021).

Research has already examined how musicians develop interpretations through practice and reflection. Héroux, (2018) investigated nine professional guitarists using recorded practice sessions, verbalizations, self-reflection questionnaires, observations, and interviews. López-Íñiguez and McPherson (2024) examined self-regulated instrumental practice through a music microanalysis protocol and practice diary reports. These studies provide precedents for investigating interpretive development and reflective learning in performance.

Practice-led research also establishes artistic practice as a means of generating knowledge (Haseman, 2006; Smith and Dean, 2009; Borgdorff, 2012; Nelson, 2013). Building on these approaches, the present study examines how poetic and dramatic constructions of solitude inform vocal decisions across four contrasting works. Its focus is the relationship among textual imagery, historical and stylistic context, and embodied vocal practice within one performer-researcher’s developing interpretations. It also explains how passage-specific observations informed intermediate interpretive understandings and the four concepts named during the final cross-case synthesis.

This issue is especially significant in vocal performance because poetic or dramatic text forms an integral component of musical interpretation. Textual meaning, syntax, prosody, pronunciation, diction, and rhetorical emphasis shape decisions concerning phrasing, breathing, resonance, vocal color, dynamics, and expressive timing. Vocal interpretation therefore emerges through the continual negotiation between musical structure, textual meaning, historical context, and embodied performance rather than from any single source of interpretive authority.

Against this background, the present study examines four contrasting vocal works by Henry Purcell, Claude Debussy, Alban Berg, and Ildebrando Pizzetti to investigate how musical solitude is interpreted through practice-led inquiry. Instead of treating solitude as a fixed expressive quality contained within either music or text, the study examines how performers transform musical and textual observations into embodied artistic decisions through iterative analysis, contextual investigation, rehearsal, recording comparison, and critical reflection.

The study is guided by the following research questions:

RQ1: How do compositional structures and poetic or dramatic texts contribute to different interpretive constructions of musical solitude in four contrasting vocal works?

RQ2: How does practice-led research enable performers to recognize, investigate, and artistically realize these different constructions of musical solitude?

Musical solitude is therefore understood not as an inherent property of the repertoire, but as an interpretive construction that emerges through the interaction of compositional organization, textual meaning, historical context, and embodied artistic practice (Taruskin, 1995). By examining how the performer-researcher notices interpretive problems, evaluates vocal alternatives, and revises decisions, the study connects reflexive rehearsal with performer learning, judgment, and self-regulation. These connections concern the processes of vocal performance, without implying that changes in self-regulatory skills were independently measured.

## Materials and methods

### Research design

This study adopts a qualitative practice-led research (PLR) design to investigate how musical solitude is recognized, interpreted, and realized through vocal performance. PLR is appropriate because interpretive decision-making develops through iterative cycles of artistic practice, contextual inquiry, experimentation, and critical reflection instead of observation alone (Borgdorff, 2012; Haseman, 2006; Nelson, 2013; Sullivan, 2010). Within this approach, rehearsal functions as the primary site of inquiry in which interpretive understanding is progressively generated, examined, and refined.

The investigation integrated four complementary modes of inquiry. First, score and text analysis identified structural, musical, poetic, and dramatic features relevant to interpretation. Second, historical and musicological contextualization situated these observations within their broader stylistic, literary, and cultural traditions. Third, embodied vocal experimentation explored alternative interpretive possibilities through rehearsal and performance. Fourth, iterative recording comparison supported the critical evaluation and refinement of successive interpretive decisions. As opposed to functioning as discrete stages, these modes of inquiry operated recursively, with observations emerging through continuous interaction between artistic practice and scholarly investigation (Cook, 2013; Leech-Wilkinson, 2009; Rink, 2002).

### Reflexive research process

Across approximately 4 months, all four works were revisited in each of 24 rehearsal sessions. Every session was recorded, producing 24 rehearsal recording files, all of which were included in the analysis. The practice record also comprised annotated scores and dated rehearsal notes, normally written after each session. Analytical attention focused on lyrics and passages that raised interpretive questions during singing, rather than on selecting a subset of recordings for inclusion. These passages prompted further inquiry into their textual, musical, historical, and stylistic significance.

Initial questions often arose from the performer-researcher’s felt response to a passage: a repeated phrase, image, or musical pattern seemed to require further interpretive attention. Subsequent score and text study and contextual inquiry helped articulate or reconsider that response and informed later rehearsal trials. Poetic imagery, syntax, prosody, and rhetoric were considered in relation to their musical setting (Proto et al., 2016).

Trials varied diction, breathing, articulation, resonance, timbre, phrasing, dynamics, and timing. Intended effects and the experience of singing were reconsidered through the performer-researcher’s recorded-listening judgments, informing whether a choice was retained, modified, rejected, or explored further. These judgments were the author’s own reflexive assessments, not independent listener evaluations.

The author’s public doctoral lecture recital, recorded on 26 June 2024 in the Recital Hall at UCSI University, provided additional evidence of vocal realization. It was considered alongside the rehearsal process, not as a substitute for that process or an independent validation of the final interpretive labels.

Recording comparison did not establish a single correct performance. It created analytical distance from singing and allowed each trial to be assessed for musical-textual coherence, historical and stylistic consistency, and performative viability (Cook, 2013; Leech-Wilkinson, 2009).

### Repertoire selection and analytical sampling strategy

The repertoire was selected through purposive qualitative sampling to generate analytically rich cases as distinct from statistical generality or stylistic typicality (Creswell and Poth, 2018; Patton, 2015). Maximum variation sampling enabled the investigation of musical solitude across contrasting compositional languages, poetic perspectives, historical contexts, and aesthetic traditions, thereby supporting analytical depth within a qualitative case-based design (Maxwell, 2013).

The selected repertoire comprises Henry Purcell’s *O Solitude*, Claude Debussy’s *Le son du cor s’afflige vers les bois*, Alban Berg’s *Wir arme Leut!* from *Wozzeck*, and Ildebrando Pizzetti’s *Oscuro è il ciel*. These works represent contrasting Baroque, Impressionist, Expressionist, and Neoclassical traditions while presenting distinct poetic constructions of solitude: affirmative withdrawal and self-reconnection in Purcell, introspection and transformation in Debussy, poverty and social alienation in Berg, and affective loneliness sustained by hope in Pizzetti. Together, these musical and textual differences created four contrasting contexts for examining how interpretive decisions emerge through the interaction of compositional structure, poetic meaning, and embodied vocal practice.

Comparison across these contrasting works supported case-grounded conceptual interpretation rather than statistical generalization or claims to represent all forms of musical solitude (Yin, 2018; Merriam and Tisdell, 2016). The analysis developed from practice-generated observations considered in relation to poetic imagery and historically situated stylistic understanding. The four final labels were not imposed on the repertoire in advance; neither was the inquiry independent of the performer-researcher’s prior knowledge or contextual interpretation.

### Reflexive analytical development and cross-case synthesis

Within each work, observations concerning particular lyrics and passages were brought together when they suggested a related poetic image or expressive concern within the work’s stylistic context. These connections provided provisional interpretive understandings that guided further exploration. In Purcell, repeated address, declamatory differentiation, and selective ornamentation were considered together in relation to contemplative engagement with solitude. In Debussy, the recurring horn gesture, changing vocal projection, and phrase release were connected through the poem’s imagery of distance and disappearance. Such groupings organized the relationship between text, musical detail, and vocal choices; they were not predefined codes applied to segmented recordings.

Cross-case comparison then considered how the different poetic and dramatic constructions of solitude called for different interpretive approaches. Purcell’s repeated invocation invited rhetorical differentiation within contemplative continuity, whereas Debussy’s distant horn imagery encouraged a less directly projected vocal presence. Berg’s dialogue required contrasts among obedience, resistance, and withdrawal; Pizzetti’s nocturnal imagery and solitary waiting called for continuity with contained expansion. Comparison therefore concerned relationships among textual meaning, stylistic context, and vocal realization, rather than the frequency of common codes or differences between historical periods alone.

Exposure, Suspension, Fragmentation, and Restraint were named at the final stage of thematic synthesis to express the broader interpretive relationships developed through these case accounts. The movement from specific observations toward broader formulations was inductive in this limited sense (Thomas, 2006), while remaining informed by textual and historical interpretation. This was a reflexive practice-led analysis, not a full grounded-theory study of the kind described by Charmaz (2014). The four terms function as case-grounded interpretive concepts rather than causal explanations or fixed categories for classifying composers and periods.

Vocal choices were evaluated through the dimensions summarized in Table 1: musical and textual coherence, historical and stylistic consistency, and performative viability. Figure 1 outlines the analytical relationships, and Table 2 retrospectively summarizes their development across the cases. Neither presents a session-by-session record or a transcript of the rehearsal notes.

**TABLE 1:** Integrated analytical dimensions applied throughout the practice-led investigation.

Analytical dimension	Analytical focus	Contribution to interpretive decision-making
Musical coherence	Musical structure, melodic contour, harmonic progression, rhythmic organization, phrase design, dynamics, articulation, and stylistic conventions	Established whether interpretive decisions remained structurally coherent and artistically consistent with the musical score.
Textual and musical coherence	Poetic meaning, semantic development, linguistic prosody, rhetorical emphasis, expressive declamation, and their interaction with musical expression	Examined how textual meaning was realized through vocal performance, enabling interpretive decisions to emerge from the continuous negotiation between literary meaning and musical structure.
Historical and stylistic consistency	Composer intentions, poetic context, historical background, stylistic conventions, and musicological evidence	Contextualized interpretive possibilities within historical and stylistic traditions while informing artistic experimentation instead of prescribing predetermined performance solutions.
Performative viability	Embodied vocal practice, breath management, resonance, timbre, articulation, expressive pacing, dramatic communication, and recording comparison	Evaluated whether interpretive decisions could be consistently embodied in performance and progressively refined through iterative rehearsal, recording-based reflection, and reflexive evaluation.

**FIGURE 1:**
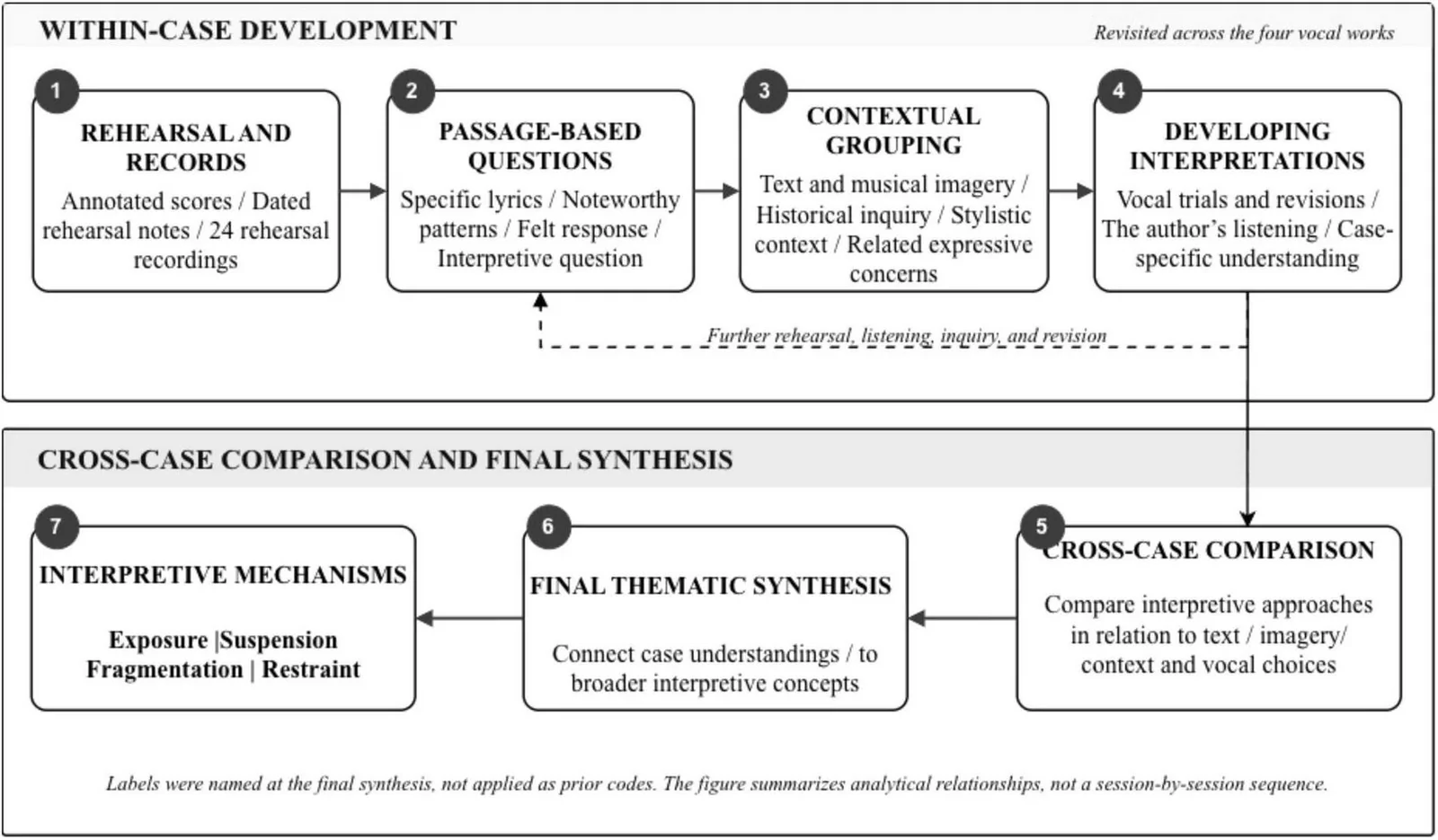
Reflexive analytical development and final thematic synthesis. Practice-generated questions are explored through text, imagery, contextual inquiry, vocal trials, and the performer-researcher’s recorded-listening judgments. Cross-case comparison concerns contrasting interpretive approaches. The four labels were named at the final synthesis, not applied as prior codes. The diagram summarizes analytical relationships, not a fixed session-by-session sequence.

**TABLE 2:** Retrospective summary of interpretive development and cross-case comparison.

Analytical focus	Henry Purcell - O Solitude	Claude Debussy - *Le son du cor s’afflige vers les bois*	Alban Berg - *Wir arme Leut!*	Ildebrando Pizzetti - *Oscuro è il ciel*
Practice-generated observation	Repeated invocation sounded static	Recurring horn gesture lost its changing distance	Continuous intensity obscured obedience and agency	Ascent sounded like an imposed climax
Interpretive problem	Renew the address without breaking contemplative continuity	Maintain a sound as its audible presence recedes	Make discontinuity structurally and dramatically coherent	Convey passing time and waiting without operatic expansion
Textual imagery and stylistic context	Philips, ground bass, Baroque rhetoric, ornament	Verlaine, Symbolist distance, French declamation	Büchner, motivic recurrence, social constraint	Leopardi, modal mixture, structural return
Retained performance strategy	Differentiated declamation; selected ornament; intimate projection	Restrained projection; lighter density; softened release	Contrasting color; paced expansion; calando withdrawal	Limited expansion; patient 5/4; inward closing color
Intermediate interpretive understanding	Renewed rhetorical address within contemplative continuity	Continuing imagined presence through acoustic recession	Changing agency across obedience, resistance, and withdrawal	Contained longing within passing time and structural return
Label named in final thematic synthesis	Exposure	Suspension	Fragmentation	Restraint

Intermediate understandings are summarized retrospectively to explain the connections among observations, textual imagery, context, and vocal choices. The wording is not presented as verbatim rehearsal-note terminology or a formal coding hierarchy. The table is an analytical synthesis, not a complete chronological audit trail. Expressive judgments refer to the performer-researcher’s own recorded-listening judgments.

### Methodological trustworthiness and reflexive positioning

Trustworthiness was assessed through transparency and coherence rather than statistical reliability or universal agreement (Lincoln and Guba, 1985; Creswell and Poth, 2018; Tracy, 2010).

Trustworthiness was supported by relating interpretive claims to score passages, texts, contextual scholarship, embodied trials, and the author’s listening to the rehearsal recordings. Dated notes and recordings supported reflection on changes in understanding, while the case accounts make the reasoning available for scrutiny. These practices address transparency and contextual coherence; they do not establish independent confirmation of the author’s judgments (Finlay, 2002; Shenton, 2004; Nowell et al., 2017).

Because the performer was also the researcher, subjectivity was treated as a situated source of insight that required explicit documentation and critical scrutiny (Finlay, 2002; Nelson, 2013; Alvesson and Sköldberg, 2018).

The unit of analysis was the author’s interpretive process and vocal performance. The author did not self-accompany. Another performer provided the accompaniment for the lecture recital, but that accompaniment supplied musical context and was not coded or evaluated as data. No audience-response or comparative-performer data were collected.

The methodological components operated as an interconnected reflexive system to the exclusion of discrete methodological protocols. Figure 2 illustrates how embodied practice, score and text analysis, historical and musicological inquiry, and recording-based critical reflection interacted recursively to inform interpretive decision-making and artistic realization throughout the investigation.

**FIGURE 2:**
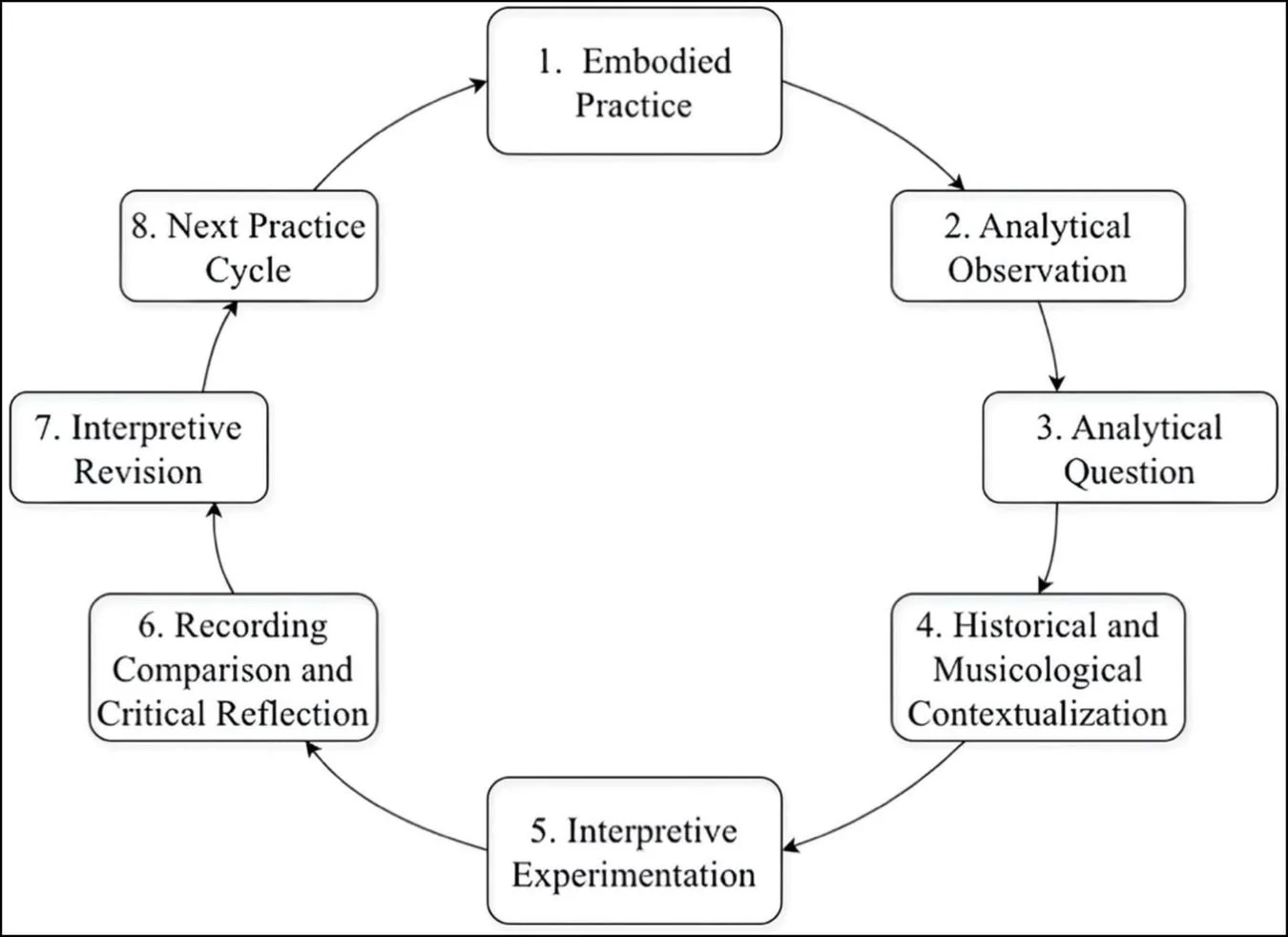
Reflexive research process used across successive rehearsal cycles.

## Results

### Overview of the reflexive analytical process

The Results are organized by case so that claims remain tied to passages, rehearsal comparisons, and retained performance decisions. Figure 1 outlines the movement from practice-generated questions to final synthesis; Table 2 summarizes the intermediate interpretive understandings and contrasts among the four cases.

Throughout these accounts, judgments about the expressive effects of rehearsal versions refer to the performer-researcher’s recorded-listening judgments, considered alongside the experience of singing and contextual inquiry.

### Comparative interpretive characteristics of the four vocal works

Although the four selected vocal works share solitude as a common thematic concern, they construct this experience through markedly different poetic, dramatic, and musical perspectives. Consequently, the interpretive challenges encountered during the practice-led investigation were shaped not only by compositional structures but also by the distinct relationships established between text, musical expression, and vocal performance (Proto et al., 2016). While the reflexive analytical process remained consistent across all four cases, each work demanded different interpretive priorities and artistic strategies. Table 3 identifies the textual orientation and principal vocal problem in each case.

**TABLE 3:** Comparative interpretive characteristics of the four vocal works.

Vocal work	Literary source	Representation of solitude	Primary interpretive orientation	Principal vocal considerations
O Solitude	Katherine Philips (after Saint-Amant)	Voluntary contemplative retreat	Rhetorical reflection	Ornamentation, rhetorical declamation, expressive intimacy
*Le son du cor s’afflige vers les bois*	Paul Verlaine	Symbolist melancholy and memory	Atmospheric suggestion	French prosody, vocal color, speech rhythm
*Wir arme Leut!*	Georg Büchner (Woyzeck)	Social alienation and psychological suffering	Dramatic realism	Emotional pacing, dramatic intensity, textual embodiment
*Oscuro è il ciel*	Giacomo Leopardi	Reflective longing and emotional solitude	Lyric introspection	Breath continuity, cantabile phrasing, expressive restraint

### Henry Purcell: *O Solitude* (from repetition to rhetorical ornamentation)

#### Textual and dramatic context

Source: Text by Katherine Philips (1632–1664), adapted from La Solitude by Marc-Antoine Girard de Saint-Amant (1594–1661) (see Table 4).

**TABLE 4:** Selected textual passages from *O Solitude.*

Original text
O Solitude, my sweetest choice!
Places devoted to the night,
Remote from tumult and from noise,
How happy here should I, and only I,
Enjoy such happiness!
No noise shall wake me from my pleasing dreams.

Unlike the other three works examined in this study, *O Solitude* presents solitude as a consciously chosen state of contemplative withdrawal as distinct from emotional suffering or social isolation. Philips’s adaptation of Saint-Amant contrasts the tranquility of retreat with the turmoil of public life, establishing the poetic perspective that informed the performer’s interpretive decisions. These textual characteristics focused the subsequent inquiry on rhetorical declamation, expressive restraint, ornamentation, and intimate vocal communication.

#### Initial musical and textual observation

Repeated rehearsal of *O Solitude* identified the recurring phrase “*O Solitude*” as the work’s principal interpretive challenge. Within the opening ten measures, the phrase appears three times and recurs nine times throughout the song, functioning not only as a compositional repetition but also as a repeated poetic invocation of solitude as a consciously embraced condition.

The phrase “O Solitude” appears three times in mm. 3–4, 7–8, and 9–10, illustrating the musical and textual repetition that initiated the performer’s analytical inquiry (see Figure 3).

**FIGURE 3:**
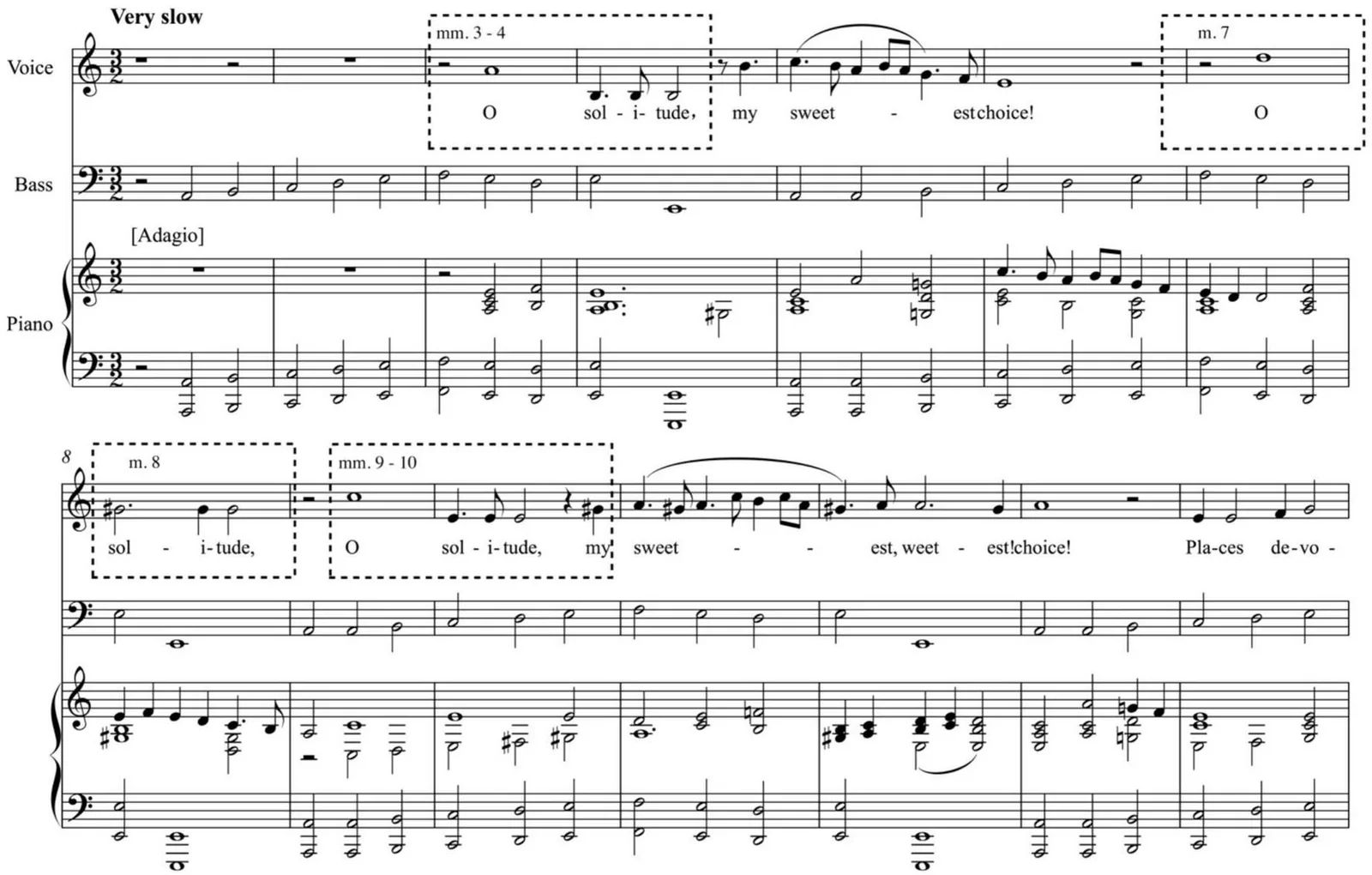
The opening fifteen measures of Henry Purcell’s *O Solitude*, mm. 3–4, mm. 7–8 and mm. 9–10 (Purcell, 1928).

During the initial rehearsals, each recurrence was performed with similar dynamics, vocal color, articulation, and phrase direction, with little use of ornamentation. Although this approach established technical consistency, comparison of successive rehearsal recordings revealed that the repeated statements became increasingly static. The interpretive problem therefore lay not in the repetition itself but in the absence of rhetorical differentiation between successive statements of the same text.

Closer examination of Philips’s poem clarified this issue. As opposed to depicting solitude as loneliness or forsakenness, the poem frames this condition as the speaker’s “sweetest choice,” juxtaposing contemplative retreat against the “tumult” and “noise” of public life. This textual perspective fundamentally altered the interpretive direction of the performance. A uniformly lamenting delivery no longer reflected the poem’s meaning. Instead, the central artistic question became how Purcell’s repeated musical setting could preserve contemplative restraint while allowing each recurrence of “*O Solitude*” to acquire renewed rhetorical significance.

#### Historical, poetic, and musicological inquiry

Purcell’s musical setting reinforces this poetic perspective through the interaction between the recurring ground bass and the comparatively flexible vocal line. While the ground bass provides structural continuity, the voice enters at different points within the repeating pattern with considerable rhythmic and melodic freedom (Holman, 1994). Repetition was therefore understood on two complementary levels: the bass established a stable contemplative environment, whereas the vocal line continually renewed the speaker’s engagement with that environment. Consequently, successive statements of “*O Solitude*” did not require identical delivery despite recurring musical material.

Scholarship on Baroque musical rhetoric similarly suggests that repetition may intensify, clarify, or redirect the expressive significance of important words instead of simply reiterating textual content (Bartel, 1997; Hatten, 2004). This perspective encouraged each recurrence of “O Solitude” to function as a renewed rhetorical utterance. Expressive differentiation was explored through subtle adjustments in consonantal emphasis, vowel duration, phrase direction, vocal color, timing, and ornamentation, allowing repeated text to acquire evolving rhetorical meaning while preserving the work’s contemplative character.

Purcell’s sensitivity to English verbal rhythm likewise placed declamation at the center of the interpretive process. The natural stress falls on solitude, while the opening “O” functions as an invocation whose expressive significance shifts according to its musical and poetic context. Successive rehearsals therefore explored the repeated phrase as a series of related rhetorical gestures, initial address, inward recognition, and quiet affirmation, without implying that the score prescribes a fixed emotional progression.

Historical studies of seventeenth-century ornamentation further demonstrated that embellishment is most effective when it clarifies cadential arrival, reinforces significant words, or renews repeated material as opposed to showcasing gratuitous vocal virtuosity (Neumann, 1978). Accordingly, each ornament was evaluated according to its harmonic placement, textual syntax, rhetorical function, and vocal viability. Ornamentation consequently became a means of strengthening the relationship between text and music instead of a standalone stylistic embellishment.

#### Interpretive revision through practice

The revised interpretive understanding was subsequently examined through successive cycles of embodied vocal experimentation. Initial exploration focused on declamation before ornamentation. Instead of introducing embellishment immediately, rehearsals varied vowel duration, consonantal clarity, phrase direction, vocal color, and emphasis on the word solitude. Comparison of successive recordings showed that excessive differentiation disrupted the work’s contemplative continuity, whereas insufficient variation reproduced the static quality of the initial interpretation. The most convincing performances maintained overall restraint while allowing each recurrence of “*O Solitude*” to acquire a distinct rhetorical inflection.

Selective ornamentation was introduced only after these declamatory possibilities had been established. Embellishments were explored primarily at cadential resolutions and phrase endings, where they were evaluated according to textual clarity, harmonic function, rhetorical purpose, and vocal viability. Through repeated experimentation, ornamentation developed a rhetorical rather than decorative function, enabling repeated words to acquire renewed expressive significance without sounding mechanically reiterated.

The poetic contrast between secluded contentment and worldly noise also informed decisions concerning dynamics and vocal timbre. Recording comparison indicated that excessive projection weakened the intimacy established by both the text and the continuo texture. Subsequent rehearsals therefore favored restrained projection, focused resonance, and speech-sensitive delivery, reserving greater dynamic expansion for moments justified by the poetic syntax or imagery.

Breathing strategy was likewise refined according to poetic syntax as distinct from musical phrase length. Maintaining complete rhetorical units preserved continuity between verbal meaning and musical expression, whereas unnecessary breaths weakened both textual intelligibility and rhetorical direction.

#### Resulting performance decision

The final performance differed substantially from the initial rehearsal approach. Repeated statements of “O Solitude” were no longer treated as identical musical events. While preserving an overall atmosphere of contemplative restraint, each recurrence was differentiated through subtle adjustments in declamation, vocal color, phrase direction, timing, and selective ornamentation. The opening statement established an intimate address, whereas subsequent repetitions progressively deepened the speaker’s relationship with solitude without developing into a conventional dramatic crescendo.

Historically informed embellishments were retained only at selected cadential points, including the resolutions surrounding mm. 28–29, m. 45, and the concluding cadence. Their purpose was not to decorate repeated material but to renew important verbal ideas, strengthen cadential arrival, and clarify rhetorical closure. Whenever an ornament obscured textual clarity, disturbed linguistic stress, or weakened the contemplative character of the work, the simpler alternative was preferred.

The resulting interpretation combined restrained dynamic pacing, clear English declamation, and subtle timbral variation. The recurring ground bass sustained the work’s contemplative stability, while the vocal line continually renewed the rhetorical significance of the repeated text through performance.

#### Interpretive development

The case brought rhetorical renewal, intimate address, and controlled differentiation into a related interpretive understanding of contemplative solitude. In the final cross-case synthesis, Exposure named the rhetorical vulnerability articulated through differentiated repetition. The term summarizes this interpretation of the work; it did not serve as *a prior* instruction for the rehearsal trials.

### Claude Debussy: *Le son du cor s’afflige vers les bois* (from motivic observation to vocal declamation)

#### Textual and dramatic context

Source: text by Paul Verlaine (1844–1896), from Poèmes saturniens (1866) (see Table 5).

**TABLE 5:** Selected textual passages from *Le son du cor s’afflige vers les bois* and English translation.

Original French text	English translation
*Le son du cor s’afflige vers les bois*	The sound of the horn grieves as it drifts toward the woods
*D’une douleur on veut croire orpheline*	With a sorrow that seems abandoned and orphaned
*Qui vient mourir au bas de la colline*	Coming to die at the foot of the hill
*Parmi la bise errant en courts abois.*	Wandering amid the winter wind in brief cries.
*L’âme du loup pleure dans cette voix*	The soul of the wolf seems to weep within this voice
*Qui monte avec le soleil qui décline*	Rising together with the declining sun
*D’une agonie on veut croire câline*	With an agony that strangely appears gentle
*Et qui ravit et qui navre à la fois.*	Enchanting and wounding the listener at the same time.

#### Initial musical and textual observation

Early rehearsals of *Le son du cor s’afflige vers les bois* initially focused on establishing technical security in the melodic line, French pronunciation, and sustained legato. As familiarity with the work increased, however, the recurring horn-like gesture introduced in the opening measures emerged as the principal interpretive question. Reappearing throughout the song, particularly in the progressively shortened and fragmented figures toward the conclusion, it prompted a central analytical question: Did these recurrences function simply as repeated musical material, or Did they represent an evolving expressive process?

At this stage, the vocal line was performed as a broadly continuous lyrical gesture. Climactic pitches received greater projection, phrases were directed toward conventional melodic high points, and relatively little distinction was made between the opening horn gesture and its later transformations. Although this approach achieved technical stability, comparison of successive rehearsal recordings showed that it failed to communicate the changing acoustic and psychological relationships suggested by both the score and the poem. The horn remained recognizable as a recurring musical idea, but its gradual transformation into distance, memory, and disappearance was not yet realized in performance.

Closer examination of Verlaine’s poem clarified this interpretive problem. The opening line personifies the horn: its sound does not simply travel toward the woods but grieves as it moves through the landscape. As the poem unfolds, the horn becomes associated with orphaned sorrow, the soul of a wolf, the declining sun, and an increasingly fragile movement toward silence. Instead of presenting a fixed musical object, the poem depicts a sound that continually changes as it recedes through space. The interpretive challenge therefore shifted from reproducing a recurring motive to communicating a poetic sound whose meaning depended upon movement, distance, impermanence, and acoustic recession.

Opening horn-like melodic gesture associated with the poem’s distant horn imagery (see Figures 4A,B).

**FIGURE 4:**
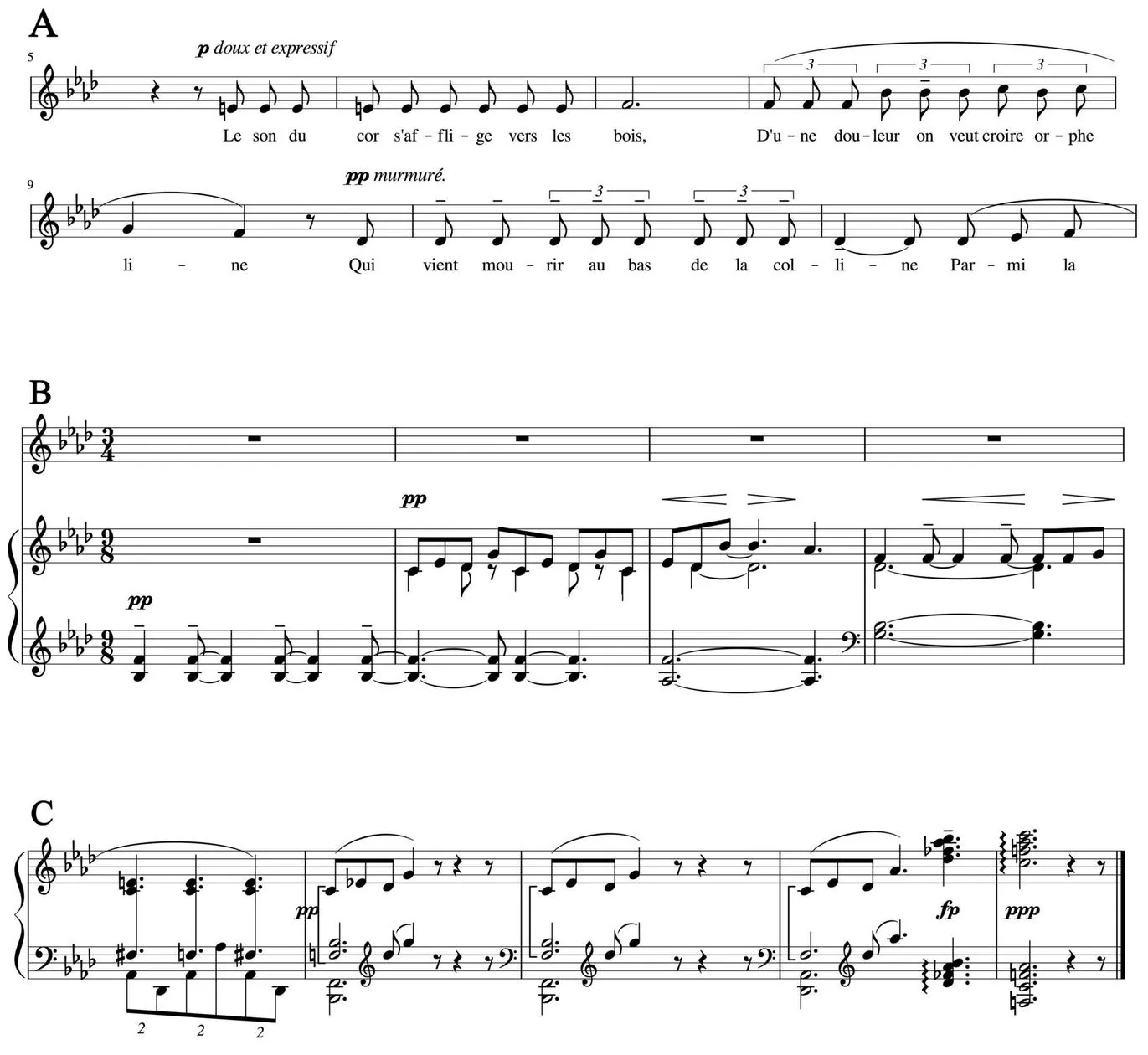
Debussy, *Le son du cor s’afflige vers les bois*: **(A)** mm. 5–11; **(B)** mm. 1–4; **(C)** mm. 35–39 (Debussy, 1901).

#### Poetic, historical, and musicological inquiry

The interpretive questions that emerged during rehearsal prompted further investigation into Debussy’s setting of Verlaine, French poetic declamation, Symbolist aesthetics, and the relationship between text and musical imagery, all of which are central to Debussy’s vocal style (Trezise, 2003; Nichols, 1992). Verlaine’s poem develops through recurring images of sound, landscape, decline, and suspended movement. Instead of constructing a linear narrative, the horn operates as both a distant acoustic presence and a mutable expressive voice, embodying shifting tonal images of grief, disappearance, and emotional ambiguity. Meaning thus arises from the interplay of sound, atmosphere, movement, and poetic suggestion, consistent with the core aesthetic tenets of French *mélodie* (Fulcher, 2005).

This poetic structure discouraged direct dramatic characterization. Instead of portraying a clearly defined dramatic figure, the singer mediates between an imagined sound and the emotional meanings gradually associated with it. Consequently, the text required clarity without rhetorical exaggeration. Although the poem repeatedly evokes grief, dying, wandering, and decline, its Symbolist imagery suggested that emotional intensity should remain embedded within the landscape, allowing expressive meaning to emerge through suggestion as distinct from explicit operatic declaration.

Debussy’s flexible declamation and gradual motivic fragmentation supported this reading. Vocal delivery had to lie between literal speech and generalized legato, responding to syntax, musical duration, and expressive direction (Trezise, 2003; Abbott, 2021).

Similarly, scholarship on French mélodie describes meaning as emerging through the continual interaction of diction, poetic structure, musical design, and vocal sound to the exclusion of reliance on any single element alone (Abbott, 2021). Declamation therefore extended beyond accurate pronunciation to include decisions concerning syllabic duration, consonantal articulation, vowel continuity, phrase release, and local emphasis, all of which shaped the relationship between poetic syntax and musical timing.

Within this context, the opening horn gesture assumed a dual interpretive function. Musically, it established the sonorous identity that unified the work; textually, it introduced the distant sound that initiates Verlaine’s poetic landscape. Reconsidering the score alongside its literary context suggested that the later fragmentation of the motive represented not merely motivic reduction but the gradual weakening of the horn’s acoustic presence. As the horn traveled toward the woods, rose with the declining sun, wandered through the wind, and finally approached silence, the score and poem revealed a shared process of progressive disappearance.

The closing measures further reinforced this interpretation. As the opening material became increasingly fragmented, the music no longer suggested a conventional climax or arrival. Instead, the horn appeared to recede beyond the performer’s immediate acoustic space, leaving only traces of its earlier presence. The principal interpretive task therefore became preserving this impression of disappearance while maintaining vocal support, textual clarity, and musical continuity.

The progressive fragmentation of the opening horn-related material contributes to the impression of increasing acoustic distance and eventual disappearance (see Figure 4C).

#### Interpretive revision through practice

This integrated textual–musical understanding prompted substantial revisions during subsequent rehearsals. French diction was no longer approached simply as accurate pronunciation but as a means of preserving the natural flow of the language within Debussy’s melodic design. Attention focused on the release of final consonants, the connection of syllables within syntactic units, and the avoidance of unnecessary emphasis on function words, allowing the poem’s principal images and verbs to emerge more clearly without disrupting musical continuity.

Phrase shaping was likewise reconsidered in relation to poetic syntax. Early rehearsals frequently directed phrases toward melodic high points, creating a series of localized lyrical climaxes. Closer examination of Verlaine’s text showed that complete poetic ideas often extended beyond individual melodic gestures, with expressive movement carried by verbs describing the horn’s changing condition rather than isolated nouns. Subsequent rehearsals therefore preserved complete syntactic units across melodic phrases, and breath points were coordinated with both musical punctuation and verbal syntax.

The word *cor* required interpretive attention because it introduced the poem’s central sound image without representing the emotional climax of every phrase. Excessive emphasis made the horn appear physically immediate, whereas Verlaine consistently situates it within an uncertain acoustic distance. A clearer consonantal onset combined with restrained vowel expansion strengthened textual definition without increasing vocal weight. Words associated with grief, dying, wandering, decline, and sighing were instead differentiated through subtle adjustments in duration, vocal color, and phrase release, allowing the horn’s gradual poetic transformation to unfold across the song.

Recording comparison subsequently informed decisions concerning dynamics, resonance, timbre, and temporal flexibility. Earlier rehearsals approached higher pitches with greater projection and resonance, but repeated listening demonstrated that excessive vocal intensity diminished the sense of spatial distance suggested by both the horn imagery and the progressively fragmented musical material. Later rehearsals therefore favored restrained dynamic expansion, focused resonance, and reduced vocal pressure, distinguishing expressive intensity from acoustic proximity while maintaining secure vocal support.

Timbral experimentation further reinforced this impression of recession. A consistently full tone made the horn’s identity appear overly stable, whereas excessive breathiness weakened French diction and interrupted musical continuity. The retained interpretation employed gradual changes in timbral density: a clear but restrained tone established the opening image, while progressively lighter vocal coloring and gentler phrase release contributed to the impression of increasing distance.

Temporal flexibility was treated with similar restraint. Excessive rubato sentimentalized the poem and weakened its broader musical continuity, whereas overly strict rhythmic control restricted the natural flow of the language. The retained interpretation employed only limited flexibility where poetic syntax, harmonic suspension, and melodic direction converged, allowing important images to emerge naturally while preserving the impression of uninterrupted movement across the landscape.

#### Resulting performance decision

The final performance differed substantially from the initial technically secure but generalized lyrical interpretation. The opening horn-related material was no longer treated as a fixed musical idea to be reproduced consistently. It established an acoustic identity whose perceived distance, timbral density, and expressive significance evolved throughout the work. The vocal line reflected this transformation through restrained projection, speech-sensitive phrasing, controlled timbral variation, and progressively softened phrase endings.

Breathing followed complete poetic and musical units wherever possible. Phrases were directed by verbs and syntactic continuations in preference to melodic peaks alone. As a result, the vocal line conveyed the horn’s movement through the landscape as distinct from presenting a sequence of isolated lyrical climaxes.

Toward the conclusion, reduced dynamic intensity and lighter timbral density were coordinated with the fragmentation of the horn-related material. The closing phrases avoided a conventional operatic resolution. Controlled decrescendo, softened articulation, and increasingly suspended phrase release allowed the sound to recede gradually, preserving the impression that it continued beyond the audible boundaries of the composition.

#### Interpretive development

The case connected acoustic recession, diminishing presence, and continuing memory through the poem’s horn imagery and the developing vocal interpretation. In the final cross-case synthesis, Suspension named the persistence of an imagined sound as its audible presence recedes. The label summarizes the relationship between poetic distance and vocal realization developed in this case.

### Alban Berg: *Wir arme Leut!* (from emotional projection to structural interpretation)

#### Textual and dramatic context

Source: Georg Büchner (1813–1837), Woyzeck, Act I, Scene 1. Passage as set by Alban Berg in Wozzeck (1925) (see Table 6).

**TABLE 6:** Selected dramatic passage from *Wir arme Leut!* and English translation.

Original German text	English translation
*Wir arme Leut! Sehn Sie, Herr Hauptmann, Geld, Geld! Wer kein Geld hat!*	We poor people! You see, Captain. money, money! If you have no money…
*Da setzt’ einmal einer Seinesgleichen auf die moralische Art in die Welt!*	How can someone like us hope to live according to the world’s moral standards?
*Man hat auch sein Fleisch und Blut!*	We too are made of flesh and blood!
*Ja, wenn ich ein Herr wär’, und hätt’ einen Hut und eine Uhr und ein Augenglas und könnt’ vornehm reden, ich wollte schon Tugendhaft sein!*	If I were a gentleman, with a hat, a watch, a monocle, and refined speech, then I too could afford to be virtuous!
*Es muss was Schönes sein um die Tugend, Herr Hauptmann.*	Virtue must indeed be a wonderful thing, Captain.
*Aber ich bin ein armer Kerl!*	But I am only a poor man!
*Wir arme Leut!*	We poor people!

#### Initial musical and textual observation

Initial rehearsals of *Wir arme Leut!* focused primarily on conveying the emotional intensity of Wozzeck’s psychological and social condition. The opening interpretation treated the passage as a sustained dramatic outburst, emphasizing vocal projection, tonal weight, and continuous emotional tension. This approach initially appeared appropriate because the text culminates in Wozzeck’s emphatic declaration, “*Wir arme Leut!”*, followed by increasingly bitter reflections on poverty, morality, labor, and social inequality.

Comparison of successive rehearsal recordings gradually revealed the limitations of maintaining a single emotional condition throughout the passage. Although the performance effectively communicated anger and desperation, continuous dramatic intensity reduced the contrast between Wozzeck’s habitual obedience to the Captain and the exceptional moments in which he articulates his understanding of social injustice. As a result, later musical and textual climaxes lost expressive impact, and the scene resembled an uninterrupted emotional outburst in place of a carefully structured dramatic progression.

Closer engagement with Büchner’s text clarified this interpretive problem. Wozzeck’s repeated “Jawohl, Herr Hauptmann” establishes a language of obedience, restraint, and social subordination, reflecting the Captain’s authority over both the conversation and Wozzeck’s social position. When Wozzeck finally speaks at greater length, his language shifts from formulaic submission to reasoned social argument, presenting poverty not simply as personal misfortune but as the condition that prevents him from meeting the Captain’s abstract expectations of morality. Within this progression, “*Wir arme Leut!*” functions as the dramatic and rhetorical center of Wozzeck’s social consciousness.

The principal interpretive question therefore shifted from how intensely Wozzeck should sing to how musical structure, textual syntax, vocal color, and dramatic pacing could reveal his progression from restrained obedience to momentary social awareness. This redirected the interpretive focus from generalized emotional projection toward a structurally informed embodiment of submission, resistance, vulnerability, and eventual resignation.

#### Historical, dramatic, and musicological inquiry

Act I, Scene 1 is organized as a sequence of stylized forms largely associated with the Captain’s discourse. Within this externally imposed structure, Wozzeck possesses little musical or verbal autonomy. His repeated “Jawohl, Herr Hauptmann” functions both as a military formula of obedience and as a recurring musical gesture that limits expressive agency. From a performer’s perspective, these restrained, repetitive responses establish the dramatic tension against which Wozzeck’s later lyrical statement acquires its expressive force.

Wozzeck’s extended response takes the form of an air and culminates in “*Wir arme Leut!*”, a melodic idea that later becomes one of the opera’s principal recurring motives (Reich, 1937). The significance of the phrase lies in the interaction between musical identity and textual meaning. Instead of conveying individual suffering alone, it articulates the shared condition of those whose poverty prevents them from fulfilling the moral expectations imposed by social authority. Its later recurrence throughout the opera consequently transforms an individual complaint into a broader expression of collective vulnerability.

This perspective fundamentally altered the desired vocal quality of the phrase. Because “Wir arme Leut!” communicates reasoned social awareness as opposed to unbridled emotional catharsis, it could not be approached simply as an anguished cry. Its broader melodic span and lyrical character briefly allow Wozzeck to articulate a coherent human perspective in contrast to the Captain’s caricatured authority. Scholarship on Wozzeck likewise challenges interpretations that reduce Wozzeck’s voice to pathology or hysteria, demonstrating that Berg’s lyricism can equally express vulnerability, solidarity, and humanity.

Recurring melodic figures in both the vocal line and orchestral texture reinforce this progression. Perle (1980) identifies these motivic relationships as fundamental to the opera’s dramatic coherence, while Jarman (1979) demonstrates that Berg’s structural procedures organize psychological development in preference to purely underpinning the drama. Reconsidering these observations during rehearsal suggested that the recurring figure in mm. 1, 7, and 13–14 functions as a structural memory whose expressive significance changes according to its dramatic and textual context.

The recurring melodic figure provides structural continuity while acquiring different dramatic functions as Wozzeck moves between obedience, social argument, and psychological withdrawal (see Figure 5A).

**FIGURE 5:**
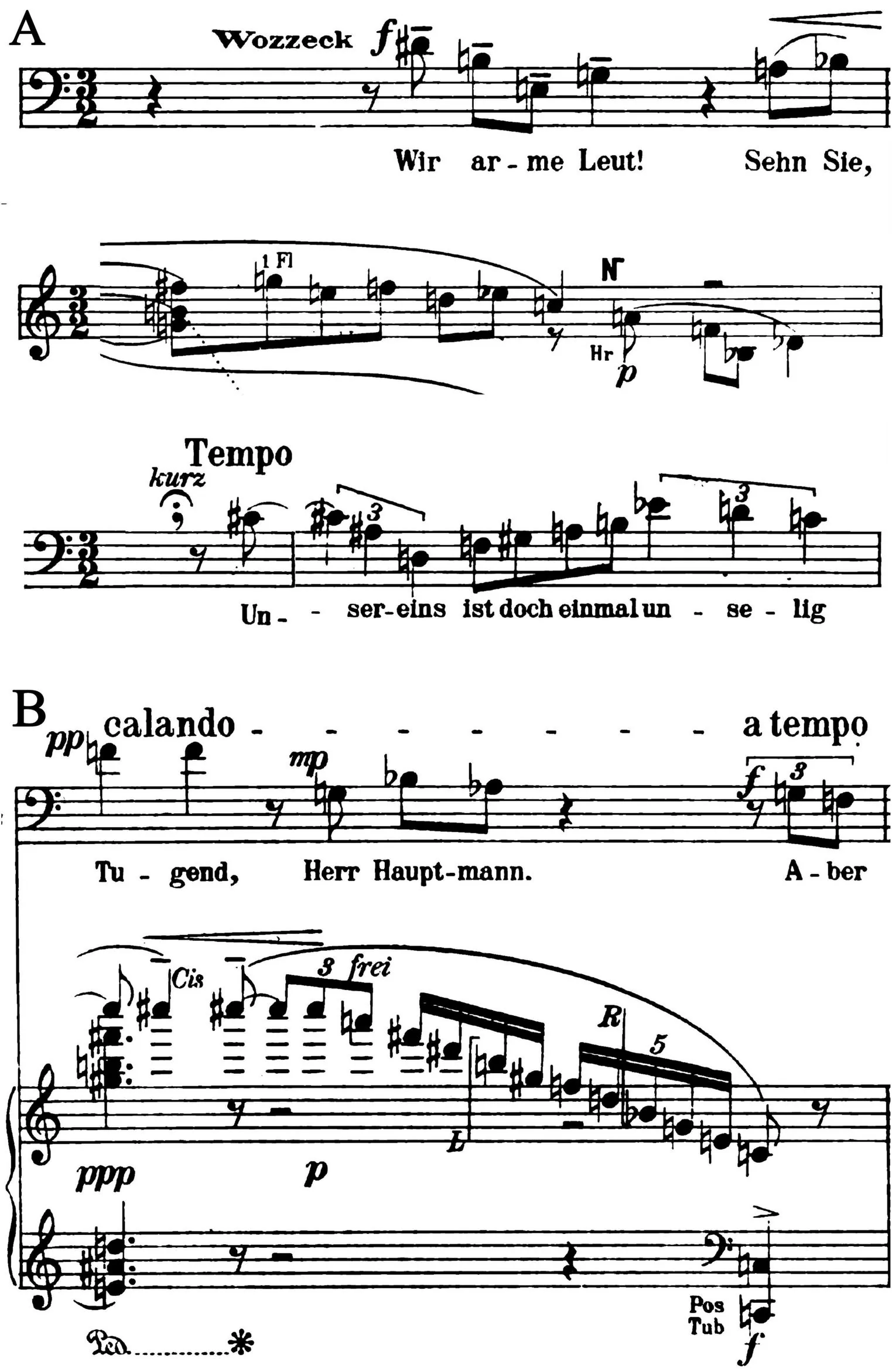
Alban Berg, “Wir arme Leut!” from Wozzeck, Op. 7. **(A)** Excerpts referred to as local mm. 1, 7, and 13–14; **(B)** excerpt referred to as local m. 12. These measure numbers are assigned by the author for the present analysis; local m. 1 corresponds to m. 136 in the full score. Excerpts from the piano reduction by Fritz Heinrich Klein (Berg, 1923).

#### Interpretive revision through practice

This revised textual and structural understanding prompted substantial changes during subsequent rehearsals. The repeated “Jawohl, Herr Hauptmann” responses were differentiated from *Wozzeck*’s later extended statements through restrained dynamics, compressed phrasing, firmer rhythmic control, and limited tonal expansion. Clear German consonants were retained, but expressive emphasis remained deliberately contained. This established Wozzeck’s externally imposed obedience while preserving vocal and dramatic energy for the gradual emergence of his social argument.

The transition into Wozzeck’s longer response was approached as a progressive expansion of verbal agency. Instead of entering with immediate dramatic force, the vocal line broadened as his thought developed. Greater legato, tonal continuity, and phrase direction distinguished his sustained discourse from the Captain’s fragmented and mannered speech. The progression toward “*Wir arme Leut!*” was therefore shaped through accumulating breath energy and increasingly focused textual emphasis.

Attention was given to the stress pattern and semantic function of “*Wir arme Leut!*” Uniform emphasis across all syllables produced an overly declamatory effect and weakened the continuity of the preceding argument. In the retained interpretation, *wir* received clear communicative emphasis because Wozzeck speaks on behalf of a broader social group, while *arme Leut* carried the principal expressive weight. Increased resonance and textual definition strengthened the phrase without obscuring its vulnerability, allowing it to communicate social awareness and personal powerlessness without becoming conventionally heroic or accusatory.

Recording comparison also prompted revisions to vocal color and weight. Initial rehearsals relied predominantly on chest-dominant production and sustained dramatic intensity. Subsequent experimentation introduced a controlled lighter color in quieter passages, particularly where the score indicates *pp*. This contrast was not used as an isolated vocal effect but to reveal moments in which Wozzeck’s social argument gives way to exhaustion, fear, and inward withdrawal.

The descending passage marked calando became particularly significant. Its reduced dynamics and diminishing momentum suggested a temporary collapse in Wozzeck’s capacity to sustain outward resistance. The line was therefore shaped through progressively reduced vocal density, softened consonantal attack, and decreasing forward pressure. A lighter vocal color was retained only when it remained integrated with the surrounding line and preserved textual intelligibility.

The descending vocal gesture, reduced dynamics, and indication calando mark a moment of expressive withdrawal (see Figure 5B). Recording comparison showed that a controlled reduction in vocal weight communicated Wozzeck’s vulnerability more effectively than uninterrupted chest-dominant projection.

Recording comparison further reshaped the overall dramatic pacing. Immediate and uninterrupted intensity weakened later climactic passages by leaving insufficient scope for further expansion. The retained interpretation therefore distinguished four interconnected dramatic conditions: constrained obedience, emerging social awareness, momentary resistance, and psychological withdrawal. These were treated as successive stages within a single dramatic trajectory rather than as separate characterizations.

Fermatas, rests, and orchestral interludes also acquired a more active interpretive function. Instead of being treated as interruptions between vocal phrases, they were understood as extensions of Wozzeck’s psychological development beyond the sung text. Silence allowed the consequences of each statement to register and prepared the altered vocal condition of the following entry; continuous visible agitation during these passages was therefore avoided.

German declamation was refined according to dramatic function. Sharper consonantal definition was retained at moments of confrontation, while Wozzeck’s reflective statements employed greater vowel continuity and a more connected vocal line. This differentiated resistance from vulnerability without fragmenting Berg’s longer melodic gestures or caricaturing the character.

#### Resulting performance decision

The final performance no longer presented *Wir arme Leut!* as a sustained dramatic outburst. Vocal intensity developed in response to the passage’s structural and textual progression. The opening exchanges with the Captain remained restrained, rhythmically disciplined, and socially deferential, while Wozzeck’s longer statements gradually expanded in breadth, resonance, and expressive continuity as his social argument emerged.

The statement “Wir arme Leut!” formed the principal expressive and structural center of the interpretation. It was delivered with focused tonal presence and clear verbal emphasis without transforming Wozzeck into a conventionally heroic figure. The phrase communicated a moment of social awareness in which he articulates the conditions governing his life while remaining unable to escape them. Expressive authority therefore emerged through insight and textual clarity in lieu of raw vocal volume.

Following this climax, vocal weight and dramatic intensity gradually receded. Wozzeck’s reflection that the poor would remain subject to labor even in heaven was conveyed through controlled pacing, reduced timbral density, and progressive withdrawal. In the calando passage, a lighter and less corporeally assertive vocal color aligned the descending musical gesture with the text’s growing resignation.

Silence and orchestral continuation were retained as integral components of the dramatic interpretation. Instead of sustaining visible agitation, these moments allowed the implications of Wozzeck’s statements to register and prepared the altered vocal condition of each subsequent entry. The performance therefore unfolded as a sequence of structurally connected psychological states to the exclusion of maintaining a static emotional state.

#### Interpretive development

Across successive rehearsal cycles, the performer’s understanding of Wir arme Leut! developed from generalized emotional projection toward a structurally informed dramatic interpretation. Musical and textual analysis revealed that recurring motives, dynamic contrasts, fermatas, orchestral interludes, and the descending calando gesture corresponded to Wozzeck’s progression from formulaic obedience to social awareness, resistance, exhaustion, and resignation.

The case linked constrained obedience, momentary agency, and withdrawal through the passage’s dramatic progression and contrasting vocal demands. In the final cross-case synthesis, Fragmentation named the divided dramatic agency articulated through changes in speech, timbre, intensity, and silence. It refers to the interpretation of *Wozzeck*’s changing condition, not simply to the presence of fragmented musical material.

### Ildebrando Pizzetti: *Oscuro è il ciel*(from dynamic control to expressive restraint)

#### Textual and dramatic context

Source: text by Giacomo Leopardi (1798–1837), adapted from Fragment 168B attributed to Sappho, set by Ildebrando Pizzetti in Due canti d’amore, No. 2 (see Table 7).

**TABLE 7:** Selected textual passages from *Oscuro è il ciel* and English translation.

Original Italian text	English translation
*Oscuro è il ciel*;	The sky is dark;
Nell’onde la luna già s’asconde	The moon has already hidden beneath the waves,
E in seno al mar le Plejadi già discendendo van.	And the Pleiades are already sinking into the sea.
È mezzanotte,	It is midnight,
E l’ora passa frattanto,	And the hours continue to pass,
E sola qui sulle piume	While I remain here alone upon my bed,
Ancora veglio ed attendo in van.	Still awake, waiting in vain.

#### Initial musical and textual observation

Initial rehearsals focused on establishing stable vocal production, melodic continuity, and the controlled dynamic shaping characteristic of Pizzetti’s restrained musical language. As technical familiarity increased, one passage repeatedly attracted analytical attention. In mm. 7–12, the vocal line rises before descending across a temporary metrical expansion from 4/4 to 5/4 and then returns to 4/4. Within the otherwise restrained musical texture, this ascending–descending contour prompted questions concerning its melodic, rhythmic, and textual significance.

Early rehearsals treated the ascending gesture as a conventional point of expressive expansion. The phrase moved toward its highest pitch through increased breath energy, fuller resonance, and a broader crescendo before relaxing into the descending line. Recording comparison, however, suggested that this familiar climactic shaping imposed a Romantic expressive profile on a passage whose poetic meaning remained considerably more restrained. Although the contour became locally prominent, its relationship to the nocturnal imagery and the song’s broader structural design remained insufficiently developed.

Closer engagement with Leopardi’s text substantially reshaped this interpretation. *Oscuro è il ciel* sets Leopardi’s expanded adaptation of an ancient fragment traditionally attributed to Sappho. The poem traces the withdrawal of the moon, the descent of the Pleiades, the arrival of midnight, and the continued passage of time while the speaker remains alone, awake, and waiting in vain. Continuous movement in the external world is therefore contrasted with an increasingly explicit condition of emotional stillness.

This relationship prompted a reconsideration of the ascending–descending contour. The ascending motion suggested a temporary expansion of perception or hope, whereas the descending line more closely reflected the gradual withdrawal of the celestial bodies. The gesture therefore did not require a fully projected climax. Its expressive meaning emerged through a brief expansion that gradually yielded to a controlled and diminishing descent.

An ascending–descending vocal contour unfolds across a temporary metrical expansion from 4/4 to 5/4 before returning to 4/4 (see Figure 6). The gesture corresponds to the poem’s imagery of celestial descent and became the principal focus of dynamic, temporal, and timbral experimentation during rehearsal.

**FIGURE 6:**
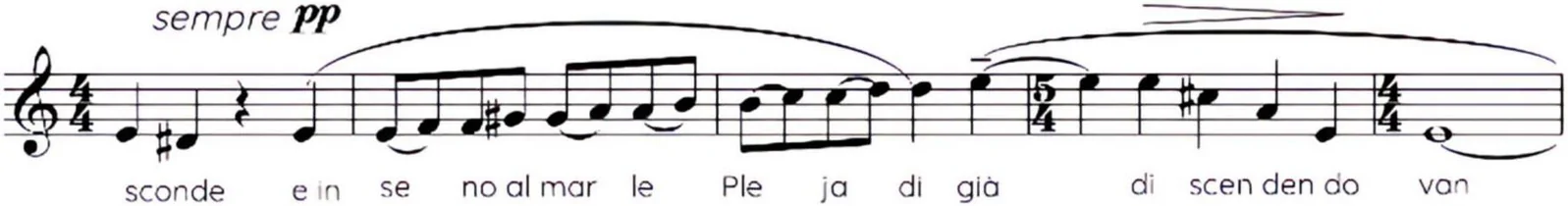
Ildebrando Pizzetti, “Oscuro è il ciel,” mm. 7–11 (Pizzetti, 2008). Musical excerpt re-engraved by the author from the cited edition and reproduced with permission.

A second observation emerged through comparison of the opening and concluding sections. Related major–minor mixtures, harmonic colors, melodic material, and phrase structures appear at both ends of the composition. Instead of signifying straightforward thematic repetition, repeated score study suggested a deliberate framing relationship. Whatley (2014) identifies this as a palindromic organization, referring to large-scale structural correspondence as opposed to lexical or poetic symmetrical patterning. The conclusion recalls the modal and harmonic language of the opening, creating a formal frame around the intervening poetic journey.

This observation generated a further interpretive question. If the opening and concluding passages share related structural materials, should they also receive comparable vocal treatment? Recording comparison demonstrated that exact expressive duplication weakened the song’s psychological progression. The opening establishes nocturnal ambiguity before the speaker’s emotional condition is revealed, whereas the conclusion follows the recognition of solitary and futile waiting. The two passages therefore required recognizable structural affinity while maintaining clearly differentiated psychological coloring.

#### Poetic, historical, and musicological inquiry

Pizzetti’s text-sensitive, modally inflected vocal style draws on earlier Italian traditions (Gatti and Waterhouse, 2001). The literary source of *Oscuro è il ciel* proved particularly significant to this interpretation. Leopardi’s adaptation expands the ancient fragment by introducing the darkened sky, the sea, the withdrawing moon, the descending Pleiades, and the speaker’s continued wakefulness. These additions establish a central temporal contrast between the continuous movement of the external world and the speaker’s suspended emotional condition.

This contrast became the principal performer-oriented interpretive framework. The song’s expressive tension arises not from static melancholy but from the coexistence of motion and immobility. Excessive rubato or dramatic expansion risks transforming the work into an operatic lament, whereas overly neutral singing weakens the cumulative emotional weight created by the passage of time. The vocal line therefore needed to communicate continuity while preserving emotional restraint.

The Italian text likewise required careful attention to syntax and declamation. The opening directs perception outward through images of the night sky and celestial movement before gradually turning inward as midnight, passing time, solitude, and futile waiting become explicit. Consequently, the song’s emotional center does not coincide with its highest melodic point but emerges through the delayed textual disclosure of isolation and unfulfilled expectation.

This textual progression also reshaped the performer’s perception of dynamic hierarchy. The opening celestial imagery required atmospheric clarity and controlled luminosity without carrying the song’s greatest expressive weight. By contrast, the concluding references to solitude, wakefulness, waiting, and futility achieved greater intensity through reduced vocal scale, narrower timbral focus, and heightened textual concentration in preference to amplified dynamic projection.

Pizzetti’s flexible use of major–minor mixture further reinforced this interpretation. As distinct from pursuing orthodox tonal closure, the modal language establishes harmonic ambiguity that evolves with the text. At the opening, it creates an atmosphere of nocturnal uncertainty; when related harmonic material returns near the conclusion, the same musical language acquires new psychological meaning following the speaker’s acknowledgment of solitude and unfulfilled waiting.

#### Interpretive revision through practice

This revised textual and structural understanding prompted a series of interpretive refinements during subsequent rehearsals. The ascending–descending contour in mm. 7–12 was first reconsidered in terms of dynamic shaping. Initial performances approached the upper pitch through a direct crescendo followed by an immediate reduction in intensity. Recording comparison showed that this imposed a conventionally Romantic climax on a passage whose poetic meaning and surrounding texture required greater restraint.

Subsequent experimentation allowed the ascending line to develop through increased directional energy without a corresponding increase in external vocal weight. Breath support intensified and forward motion was maintained, while dynamic expansion remained proportionate. As the line descended, vocal weight was gradually released through reduced timbral density and forward pressure, allowing the image of celestial descent to emerge through diminishing presence rather than an overt decrescendo.

The temporary metrical expansion from 4/4 to 5/4 was similarly reconsidered as an enlargement of musical time. Early rehearsals prioritized metric precision, producing a stable but comparatively square phrase. The retained interpretation allowed the additional beat to function as a subtle temporal expansion within the broader flow. Tempo remained essentially unchanged, but sustained breath support and patient textual delivery enabled the phrase to unfold without isolating the metrical change as a separate dramatic event.

Italian declamation became increasingly central to the interpretive process. Early emphasis on sustained vowels preserved legato but occasionally obscured the movement of the poetic syntax. The revised interpretation maintained vowel continuity while giving greater definition to verbs such as *s’asconde, discendendo, passa, veglio, and attendo*. These verbs articulate the poem’s temporal contrast between celestial movement, passing time, and continued wakefulness.

Attention was given to *discendendo van.* Because the text describes continuous downward movement rather than sudden descent, the phrase was shaped through connected vowels, restrained consonantal articulation, and gradually diminishing timbral intensity. Excessive accentuation fragmented the image, whereas overly smooth legato weakened textual clarity. The retained version balanced uninterrupted melodic continuity with precise verbal articulation.

The transition into “*È mezzanotte*” marked a shift from external observation to explicit awareness of passing time. Strong accentuation made the moment sound unduly revelatory, while overly restrained delivery weakened its structural significance. A focused but contained tone allowed the phrase to register as recognition rather than shock.

The following words, “*e l’ora passa frattanto*,” required a similar balance. Excessive rubato contradicted the text by suspending the very sense of movement it describes, whereas rigid tempo diminished the painful awareness of time’s continuation. The retained interpretation preserved forward momentum while introducing a slight agogic emphasis on *passa*, acknowledging the passage of time without disrupting the larger phrase.

The concluding lines, “e sola qui sulle piume/ancora veglio ed attendo in van,” gradually became the psychological center of the interpretation. Initial performances increased dynamic intensity at this point, but recording comparison showed that greater projection externalized the speaker’s emotional condition and reduced the intimacy of the text. Subsequent rehearsals therefore employed a reduced dynamic scale, narrower timbral focus, and more concentrated verbal delivery.

The word sola received sufficient emphasis to disclose isolation without becoming an independent exclamation. It initiated a syntactic and emotional progression toward *veglio, attendo*, and finally in van: the speaker is alone, remains awake, continues to wait, and recognizes the futility of that waiting. The final words were shaped through controlled dynamic withdrawal, clear diction, and continuous breath support, maintaining vocal presence while gradually relinquishing expectation.

The palindromic relationship between the opening and concluding sections also influenced breath organization, vocal color, and phrase proportion. Corresponding passages retained comparable dynamic contours and related breath planning so that the return of the opening major–minor language remained perceptible. The conclusion, however, adopted a more inward timbre, reduced expansion, and less externally directed phrase release. This preserved the structural frame while communicating the psychological transformation produced by the intervening text.

#### Resulting performance decision

The final performance preserved the song’s melodic continuity, harmonic ambiguity, and structural balance while avoiding excessive dynamic expansion. The ascending portion of the central contour developed through increasing directional energy without becoming an operatic climax. Its descent was shaped through a gradual reduction in timbral density and forward motion, aligning the vocal gesture with the downward movement of the moon and Pleiades.

Dynamic contrasts were distributed proportionately across the work. The opening nocturnal imagery was conveyed through contained luminosity, focused resonance, and clear Italian diction. “*È mezzanotte*” introduced greater concentration without a substantial increase in volume, while the final disclosure of solitary waiting carried the greatest psychological intensity through a reduced vocal scale and heightened textual focus.

Breath organization followed both musical structure and poetic syntax. Longer units were maintained where the text described continuous visual or temporal movement, particularly in the references to celestial descent and passing time. In the final section, phrasing preserved the semantic progression from solitude to wakefulness, continued waiting, and the recognition of futility.

Vocal color gradually shifted from outward observation toward inward concentration. The opening retained sufficient resonance to establish the nocturnal setting, while later phrases adopted a narrower and more intimate timbral profile. This transition emerged through controlled adjustments in resonance, consonantal energy, vowel expansion, breath pressure, and phrase release rather than abrupt changes in vocal production.

The opening and concluding sections remained recognizably related through comparable phrase proportions, dynamic scale, and breath planning. Their expressive functions, however, were differentiated. The opening major–minor mixture established nocturnal ambiguity before the speaker’s emotional condition was disclosed; its return near the conclusion followed the recognition of solitary and futile waiting. The final passage therefore recalled the opening structurally while carrying a transformed psychological meaning.

#### Interpretive development

Across successive rehearsal cycles, the performer’s understanding of *Oscuro è il ciel* developed from localized dynamic control toward an integrated interpretation of poetic time, celestial movement, structural correspondence, and expressive restraint. Musical analysis identified the ascending–descending contour, temporary metrical expansion, modal mixture, and relationship between the opening and concluding sections. Textual analysis clarified that these features operate within a poem structured around the contrast between a moving nocturnal world and a speaker immobilized by unfulfilled expectation.

The case connected contained expansion, passing time, structural return, and measured withdrawal through the contrast between celestial movement and solitary waiting. In the final cross-case synthesis, Restraint named the contained longing articulated through continuity, proportion, and controlled vocal expression. It describes an active interpretive choice rather than emotional neutrality.

### Cross-case synthesis of interpretive development

Comparison focused on how each work’s textual construction of solitude called for a distinct vocal approach. Purcell’s repeated address was differentiated within contemplative continuity; Debussy’s horn imagery was realized through acoustic recession and speech-sensitive phrasing. Berg’s dialogue required changes in vocal intensity and agency, while Pizzetti’s nocturnal imagery and waiting called for sustained movement with contained expansion. The final labels (Exposure, Suspension, Fragmentation, and Restraint) condense these relationships between textual meaning and vocal realization. Their distinction rests on the case-specific interpretive reasoning, not on assigning one mechanism to each historical style.

Figure 7 brings together the repertoire-specific features and vocal choices summarized by these labels, while Table 2 presents the intermediate understandings and basis for comparison.

**FIGURE 7:**
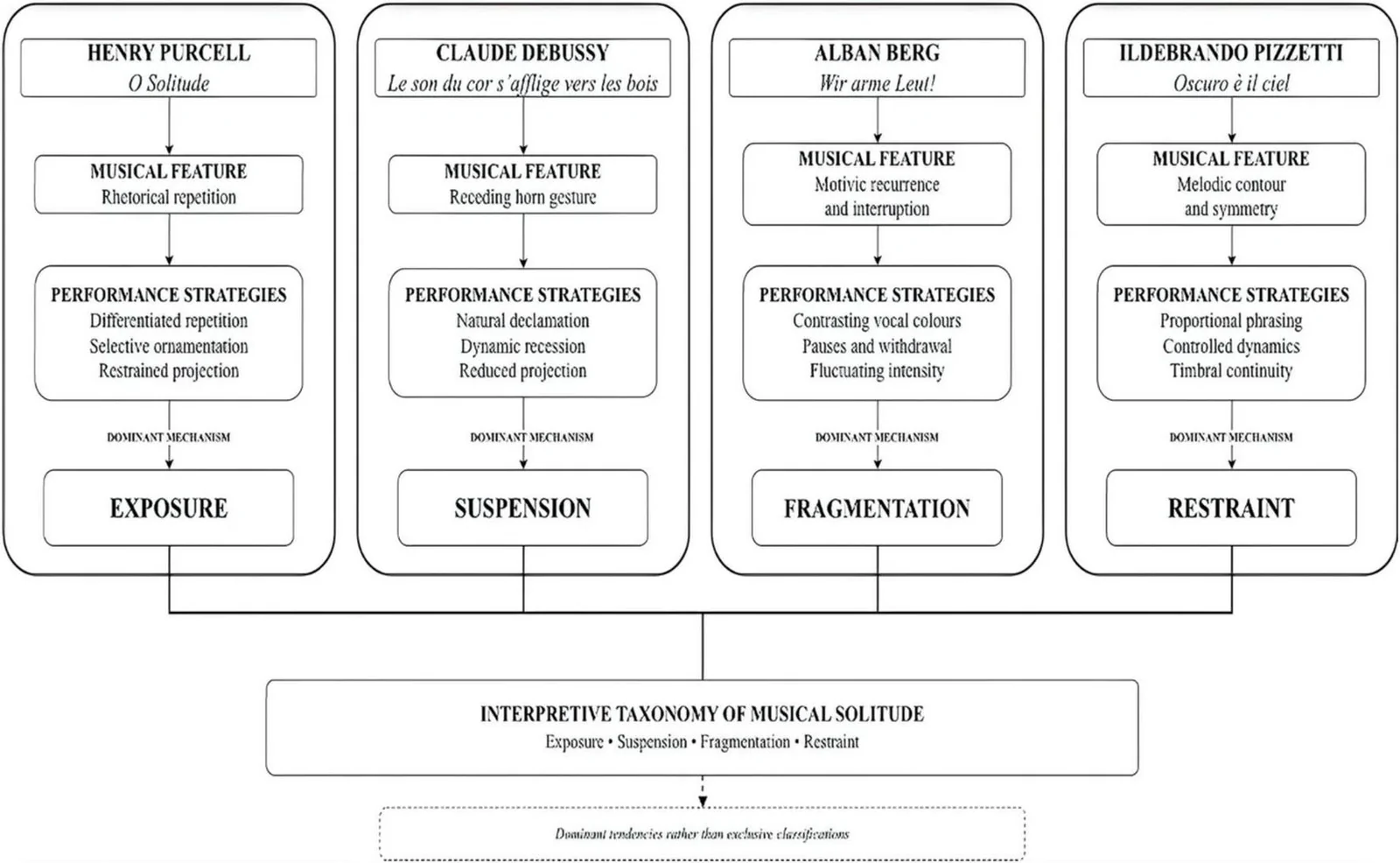
Four vocal works and four interpretive mechanisms of musical solitude.

## Discussion

### From musical structure to musical meaning: reconstructing solitude as an interpretive mechanism

The four cases suggest that musical solitude did not arise from compositional devices or texts alone. It became interpretable through relationships among score, language, context, and embodied trial, consistent with hermeneutic accounts of meaning as dialogue rather than retrieval (Gadamer, 2004; Davies, 2001; Levinson, 1993).

The same device could serve different meanings. Purcell’s repetition supported renewed disclosure; Debussy’s horn motive receded; Berg’s recurring figures organized divided agency; and Pizzetti’s structural return contained longing. The mechanisms therefore name interpretive relationships, not intrinsic musical signs (Hatten, 2004).

Accordingly, Exposure, Suspension, Fragmentation, and Restraint remain case-level formulations: rhetorical vulnerability, unresolved acoustic distance, divided dramatic agency, and proportionally contained longing.

### Practice-led research as reflexive analytical inquiry

Practice generated questions that score study alone had not resolved. Rehearsal exposed static repetition, misplaced climax, undifferentiated intensity, and excessive projection; contextual inquiry and further trials then altered those choices. Practice therefore functioned as evidence-generating inquiry (Borgdorff, 2012; Haseman, 2006).

Recordings created distance between producing and evaluating sound. Choices that felt persuasive while singing sometimes appeared exaggerated, structurally disconnected, or textually unclear on replay; those discrepancies prompted further trials.

The resulting claims are neither performer-independent discoveries nor unexamined impressions. They are situated judgements supported by a documented sequence of observations, comparisons, and revisions (Borgdorff, 2012; Smith and Dean, 2009).

### The four-part interpretive taxonomy: an analytical vocabulary for musical solitude

The taxonomy arose from a final thematic synthesis of the developing case interpretations. Within each work, particular observations were connected through poetic imagery and stylistic context; comparison then clarified how those connections called for different vocal approaches. The four labels summarize these interpretive relationships without implying that each appeared equally across all cases or that the analysis established a formal coding hierarchy.

Exposure names differentiated rhetorical vulnerability; Suspension, maintained acoustic and temporal distance; Fragmentation, organized psychological and dramatic discontinuity; and Restraint, proportionate containment of expressive resources.

These labels do not classify composers, techniques, or fixed emotions, and they do not exhaust possible forms of musical solitude. Other performers or repertoires may produce different categories (Flyvbjerg, 2006; Tracy, 2010).

The taxonomy is a case-bounded analytical vocabulary. Its broader value depends on whether later studies can usefully test, alter, or reject it.

### Methodological contribution: making practice-led inquiry transparent

The methodological contribution is an explicit account of how practice-generated questions, textual and contextual inquiry, vocal experimentation, and recorded-listening judgments informed the final interpretations. Figure 1 and Table 2 summarize this reasoning rather than reproduce a complete raw-data audit trail. This offers a situated account of practice-led inquiry, not a new universal method (Biggs and Büchler, 2008; Candy and Edmonds, 2018).

Transparency does not standardize interpretation. Other performers may follow a comparable documentation process and reach different decisions; that possibility is part of the framework’s testability (Lincoln and Guba, 1985; Tracy, 2010).

### Limitations, transferability and future directions

The study is limited to four Western vocal works and one performer-researcher. It documents one interpretive trajectory, not universal categories or the responses of other performers and listeners (Sullivan, 2010).

The study did not compare performers or collect systematic audience responses. Recording comparison supported the author’s reflexive evaluation but cannot establish how listeners perceived the four mechanisms. The final labels are retrospective interpretive formulations; the published case summaries do not reproduce the full rehearsal chronology.

Further studies could test or adapt the analytical pathway with multiple performers, other musical traditions, and qualitative audience response. Such work could determine which categories remain useful and which require replacement (Clarke, 2005; Cook, 2013; Leech-Wilkinson, 2009).

## Conclusion

Across four vocal works, musical solitude became meaningful through the interaction of score, text, context, and embodied trial. Rehearsal evidence remained repertoire-specific: rhetorical renewal in Purcell, acoustic recession in Debussy, divided agency in Berg, and contained expansion in Pizzetti.

The final thematic synthesis named these case-grounded interpretive relationships Exposure, Suspension, Fragmentation, and Restraint. They developed from practice-based questions explored through text, imagery, historical context, and vocal experimentation, rather than from a predefined classification of musical solitude.

The study offers a transparent, case-bounded pathway from annotated scores, notes, and recordings to interpretive claims. That pathway and its labels may be tested or adapted in further studies; the present evidence does not establish their validity across repertoires or performers.

## Data Availability

The raw data supporting the conclusions of this article will be made available by the author, without undue reservation.
